# Mapping current evidence and knowledge gaps in gestational diabetes mellitus in Vietnam and Vietnamese diaspora: a scoping review

**DOI:** 10.1080/16549716.2026.2734701

**Published:** 2026-09-28

**Authors:** Chau T.N. Tran, David G. Popovich, Carl Lachat, Rajaraman Eri, Hanh T.M. Tran, Tuyen Truong

**Affiliations:** a School of Science, STEM College, RMIT University, Melbourne, Australia; b School of Science, Engineering and Technology, RMIT University, Ho Chi Minh City, Vietnam; c Department of Food Technology, Safety and Health, Faculty of Bioscience Engineering, Ghent University, Ghent, Belgium; d Department of Nutrition and Dietetics, Hoan My Sai Gon Hospital, Ho Chi Minh City, Vietnam

**Keywords:** Gestational diabetes, Vietnamese, GDM, prevalence, risk factors

## Abstract

Gestational diabetes mellitus (GDM) is an increasing public health concern among Vietnamese women. However, evidence across Vietnam and Vietnamese diaspora populations remains fragmented. Reported in accordance with the PRISMA-ScR guideline, this scoping review mapped available evidence on GDM among Vietnamese-origin women from database inception to November 2025 to inform future research and culturally responsive care. English and Vietnamese literature was searched across international databases, national repositories, and grey literature. Seventy-six eligible studies were included and synthesised descriptively. In Vietnam, the sample-size-weighted average prevalence of GDM was 23.24%, with substantial variation between 2010 and 2024. Consistent candidate risk factors included advanced maternal age, higher pre-pregnancy body mass index, family and obstetric history, diet-related factors, and physical inactivity. Studies also reported limited knowledge among pregnant women. GDM was associated with adverse maternal outcomes, larger birthweight, whilst maternal knowledge remained limited. Meanwhile, studies concerning the Vietnamese diaspora consistently reported a higher GDM prevalence and related obstetric complications compared with host-country populations. However, diaspora research lacked interventional trials. Existing literature is heavily hospital-based and methodologically heterogeneous. Future research must prioritise standardised screening protocols, community-based longitudinal cohorts, and controlled interventions to improve holistic GDM prevention and management across both Vietnam and migrant healthcare settings.

## Statement of Significance

**Problem or Issue:**

Gestational diabetes mellitus (GDM) is increasing public health concern for Vietnamese women globally. Domestic incidence continues to rise despite screening efforts, whilst diaspora populations face a disproportionately higher GDM burden and severe long-term metabolic risks compared to host populations.

**What is Already Known:**

Existing studies are fragmented. Domestic research is predominantly hospital-based, whilst diaspora studies focus strictly on disease prevalence and lack interventional data. No comprehensive synthesis has been available to inform maternity care practice or provide an overall evidence perspective.

**What this Paper Adds:**

This scoping review synthesises 76 English- and Vietnamese-language studies on GDM in Vietnam and the diaspora. It identified major gaps in community-based surveillance, standardised diagnostic reporting, longitudinal maternal and offspring outcomes, psychosocial research, controlled interventions, postpartum diabetes prevention, and culturally adapted care for Vietnamese-origin women globally.

## Paper Context

- **Main findings**: Gestational diabetes mellitus is a substantial and rising maternal health burden among Vietnamese women in Vietnam and diaspora settings. Reported risk factors included older maternal age, higher pre-pregnancy BMI, family and obstetric history, diet, and physical inactivity.
- **Added knowledge**: This review mapped 76 English- and Vietnamese-language studies into the first comprehensive evidence map of gestational diabetes mellitus in Vietnamese‑origin women and identified key gaps in longitudinal, behavioural, intervention, and biological research.
- **Global health impact for policy and action**: The findings support standardised care pathways, culturally appropriate counselling, and stronger postpartum follow-up in Vietnam and other low- and middle-income and migrant health settings.

## Background

GDM is a pregnancy-related condition of glucose regulation that typically develops in the second or third trimester and resolves after giving birth. Poor glycaemic control is associated with adverse maternal and neonatal outcomes, including pre-eclampsia, macrosomia, caesarean birth [1], and an increased long-term risk of type 2 diabetes in both mothers and their offspring [2].

In Vietnam, GDM has become a growing public health concern. Hospital-based surveillance across specialised obstetric facilities indicates that detection rates increased from approximately 3–4% in 2004 to around 20% of screened pregnant women by 2017. In Ho Chi Minh City, the two largest tertiary maternity hospitals (Tu Du and Hung Vuong) have reported detection rates approaching 20% in recent years, based on large-scale annual screening [3].

In response, the Ministry of Health of Vietnam issued National Guidelines for the Prevention and Management of GDM in 2018, with an updated version in 2024, informed by multidisciplinary expertise and international recommendations [3,4]. Despite these efforts, challenges remain in early detection and equitable access to nutrition counselling and lifestyle interventions, particularly in resource-limited settings.

Vietnam also provides an important case for global health learning because evidence on GDM extends beyond women living in Vietnam. Vietnamese diaspora populations in high-income countries have often been reported to experience a higher burden of GDM than host populations, suggesting that ethnicity, migration, culture, diet, and health-system context may interact to shape risk and care [5–10]. Synthesising evidence from both Vietnam and diaspora settings can therefore inform culturally appropriate screening, counselling, and follow-up strategies not only for Vietnamese women, but also for other migrant and high-risk populations.

Existing evidence on GDM among Vietnamese women is fragmented across study designs, clinical settings, diagnostic criteria, and publication sources, with many studies published in Vietnamese and less accessible internationally. Given this breadth and heterogeneity, a scoping review was appropriate to map the extent and nature of available evidence, rather than to produce pooled estimates. This review asked: what is known about GDM among Vietnamese-origin women in Vietnam and diaspora communities regarding prevalence, candidate risk factors, knowledge, maternal and neonatal outcomes, interventions, and evidence gaps? It aimed to synthesise evidence from inception to November 2025 and inform culturally tailored strategies for future research, maternity care, and policy.

## Methods

The review protocol was developed a priori as part of a doctoral research project, subjected to institutional academic review, and formally registered on the Open Science Framework (https://osf.io/vcgfm). As this scoping review used secondary data from published literature, ethics approval was not required.

### Inclusion criteria

Studies were included based on the Population–Concept–Context framework to ensure a transparent and systematic selection process [11].

Population: Studies included pregnant women of Vietnamese origin in Vietnam or diaspora settings, including those with GDM. Vietnamese origin was defined by the primary study, mainly through maternal birthplace or self-reported ethnicity/ancestry. Studies of non-Vietnamese or non-pregnant populations, pre-existing type 1 or type 2 diabetes, or animal models were excluded.
Concept:
  - This review mapped the extent and nature of GDM evidence. To accommodate evolving guidelines, studies using any validated national or international diagnostic criteria were included. Eligible concepts included epidemiological metrics, candidate determinants (demographic, clinical, lifestyle, dietary, and emerging biological risk factors), women’s knowledge and perceptions of GDM, and the evaluation of interventions for glycaemic control.
  - Primary outcomes included the identification of patterns, associations, and existing research gaps within the current body of evidence.

Context: Studies conducted across all settings where GDM is managed or studied, including specialized clinical facilities and community or field settings, were included. No restrictions were applied regarding geographic regions, cultural dietary practices, or publication time frames (from inception to November 2025). Only literature published in English or Vietnamese was considered.

### Types of evidence sources

This scoping review included a broad range of evidence sources to capture the full scope of research on GDM among Vietnamese-origin women. Eligible study designs included observational studies (prospective/retrospective cohorts, case-control, cross-sectional), quasi-experimental studies, qualitative research exploring maternal experiences and behaviours, and relevant grey literature (e.g. reports, national guidelines). Systematic reviews, narrative reviews, meta-analyses, and single case reports were excluded to maintain the focus on mapping primary population-level evidence.

### Data sources and search strategy

A comprehensive literature search was conducted across international databases, including PubMed, Scopus, Web of Science, Cochrane Library, Google Scholar, Embase, ClinicalTrials.gov, and ProQuest, as well as Vietnamese databases and journals, including Journal of Obstetrics, Vietnamese Journal of Medicine, Ho Chi Minh City Journal of Medicine, Can Tho Journal of Medicine and Pharmacy, Journal of Medical Research, Journal of Nutrition and Food, Journal of Preventive Medicine, Journal of Clinical Medicine and Pharmacy.

Additional searches were conducted in Vietnamese national medical repositories, institutional databases, and reference lists of included studies to identify grey literature, including reports and non-peer reviewed publications.

The search strategy was structured using the PCC framework to ensure systematic retrieval of relevant literature. A standardised search syntax was developed and systematically applied across all databases: ‘Diabetes, Gestational’ OR (‘gestational’ and ‘diabetes’) OR (‘diabetes’ AND ‘pregnancy induced’) OR ‘GDM’ OR ((‘Pregnancy’ OR ‘Gestation’ OR ‘Pregnant’) AND (‘Glucose Tolerance’ OR ‘OGTT’)) AND (‘Vietnam’ OR ‘Vietnamese’ OR (‘Viet Nam’). All records were managed in EndNote Reference Manager 21, which was used for deduplication and organisation throughout the review process.

Supplementary searching included backward citation tracking of relevant reviews and meta-analyses excluded as secondary evidence sources.

### Study selection

Two independent reviewers (C.T.T.N. and P.D.G.) screened titles, abstracts, and full texts against the predefined Population-Concept-Context eligibility criteria. Discrepancies were resolved by consensus.

Eligible studies included peer-reviewed articles and grey literature addressing GDM among women of Vietnamese origin, published in English or Vietnamese. Studies focusing exclusively on non-Vietnamese populations, lacking full text, or classified as case reports or reviews were excluded (Figure 1).

**Figure 1.**
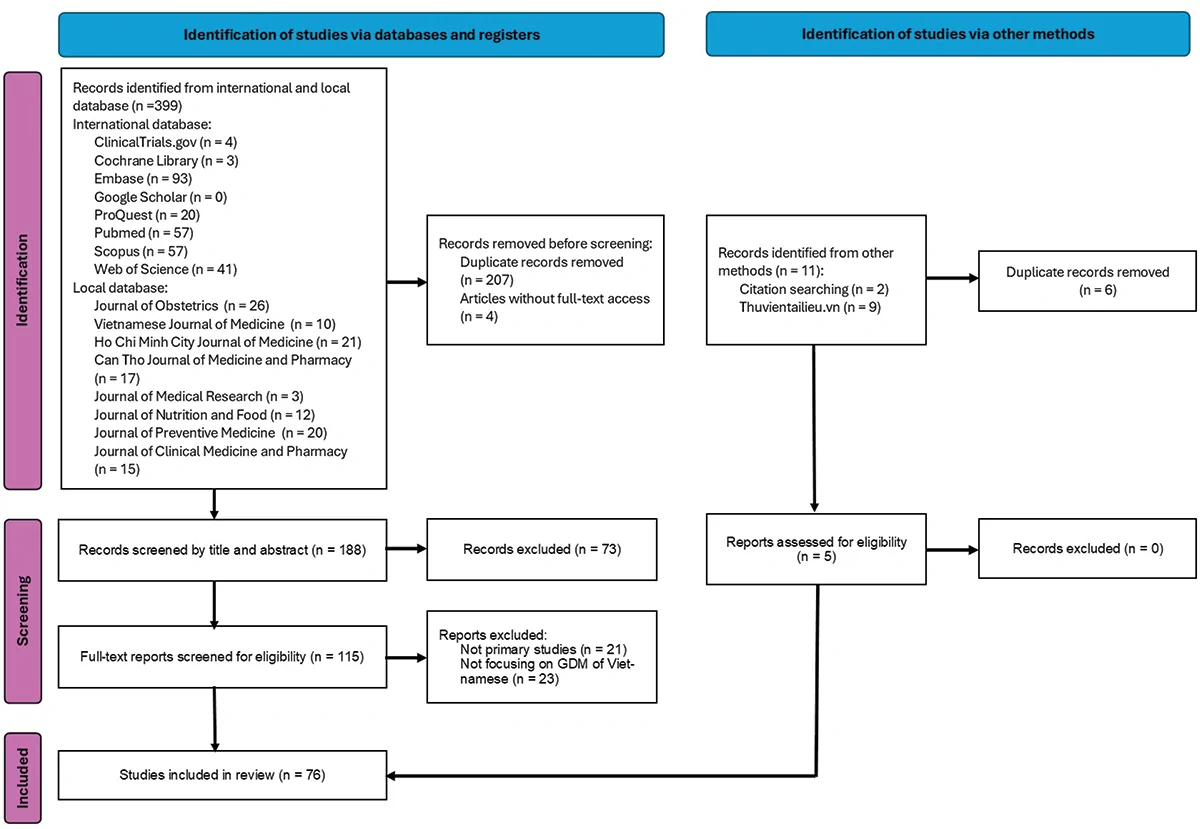
PRISMA flow diagram of study selection on gestational diabetes mellitus in Vietnamese women.

### Data extraction

Data were extracted independently by two reviewers (C.T.T.N. and P.D.G.) using a predefined, piloted data extraction form capturing study characteristics, GDM prevalence, risk factors, maternal and neonatal outcomes, and intervention details. The form was pilot tested on five studies and refined prior to full extraction. Discrepancies were resolved by consensus.

Extracted data were systematically summarised in Tables 1–4, including study design, setting, population characteristics, sample size, number of GDM cases, and reported outcomes.

**Table 1.** GDM prevalence among Vietnamese migrant women compared with other ethnic groups and host-country populations.

Authors, year	Diagnostic criteria	Population Studied
(Bastola et al., 2021) [5]	Finnish Current Care Guidelines, 75 g OGTT, ≥1 value abnormal: Fasting plasma glucose ≥ 5.3 mmol/l 1-hour value ≥ 10.0 mmol/l 2-hour value ≥ 8.6 mmol/l	Finnish origin: 8.7%, Vietnamese: 9.2%
(Beischer et al., 1991) [6]	The 1985 World Health Organization Criteria, 75 g OGTT, ≥1 value abnormal Fasting plasma glucose > 7.8 mmol/l 2-hour value > 12.2 mM	Overall: 5.5%, Vietnamese: 7.3%
(Cheng et al., 2015) [7]	Not available	Asian: 6.8%, Non-Hispanic White: 2.6%, Vietnamese: 7.0%
(Chu et al., 2009) [8]	Not available	White: 3.8%, Black: 3.5%, Hispanic: 3.6%, Vietnamese: 6.2%
(Kim et al., 2013) [9]	Not available	Overall: 7.8%, Vietnamese: 13.0%
(Shah et al., 2021) [10]	Not available	General (2019): 6.35%, Vietnamese (2019): 10.92%

Note: OGTT Oral Glucose Tolerance Test.

**Table 2.** GDM prevalence among Vietnamese-origin women according to maternal country of birth.

Authors, year	Diagnostic criteria	GDM Prevalence
(Henry et al., 1993) [12]	50 g OGTT, a combination of 2 value abnormal 1-hour value ≥ 9 mmol/l 2-hour value ≥ 7 mmol/l	Born in Vietnam (7.8%) vs Born in Australia (4.3%)
(Shah et al., 2022) [13]	Not available	General population: Born outside (7.03%) vs. in the U.S. (5.32%) Vietnamese subgroup: Born outside (10.01%) vs. in the U.S. (7.71%)
(Sullivan & Shepherd, 1997) [14]	75 g glucose load administered after an overnight and two hours after 75 g OGTT, ≥1 value abnormal Fasting plasma glucose ≥ 5.5 mmol/L 2-hour value ≥ 8 mmol/L	Born in Vietnam: 5.3%; Born in Australia: 1.6%
(Dahlen et al., 2013) [15]	Not available	Total population: Non-Australian-born (7.5%) vs. Australian-born (3.1%) Non-Australian-born subgroup: born in Vietnam (11.4%)

Note: OGTT Oral Glucose Tolerance Test.

**Table 3.** Summary of hospital-based studies on gestational diabetes mellitus prevalence among local Vietnamese pregnant women.

Authors, year	Regions	Provinces/ Cities	Population	Inclusion	Exclusion	Diagnostic criteria	Study design	Start Year	End year	Total number of participants	Number of GDM	GDM Prevalence
(Do & Nguyen, 2018) [16]	Central	Hue	Pregnant women at the Obstetrics Clinic, Hue University of Medicine and Pharmacy Hospital from January to December 2017	24–28 weeks of gestation	Having conditions affecting glucose metabolism Using medications affecting glucose metabolism Acute illnesses Participation in other studies or loss to follow-up during the study period. Completing pregnancy at other facilities	IADPSG	Prospective cohort	2017	2017	575	51	8.9%
(Duyen & Thuong, 2019) [17]	South	Ca Mau	Pregnant women at Ca Mau Obstetrics and Paediatrics Hospital from October 2017 to March 2018	24–28 weeks of gestation Singleton pregnancy	Pre-existing diabetes before pregnancy Suffering from malignant diseases, severe medical conditions, cardiovascular diseases, mental disorders Foetal congenital abnormalities. Using medications affecting glucose metabolism	IADPSG	Cross-sectional	2017	2018	260	55	21.2%
(Hang et al., 2016) [18]	South	District 5, Ho Chi Minh City	Pregnant women at An Binh Hospital, Ho Chi Minh City from August 2015 to January 2016	≤28 weeks of gestation	Pre-existing diabetes before pregnancy. Having conditions affecting glucose metabolism	IADPSG	Cross sectional	2015	2016	465	63	13.5%
(Hien et al., 2025) [19]	North	Quang Ninh	Pregnant women at Vietnam – Sweden Uong Bi Hospital, Quang Ninh, from July 2023 to July 2024	24–28 weeks of gestation	Having conditions affecting glucose metabolism Using medications affecting glucose metabolism Acute illnesses	IADPSG	Cross sectional	2023	2024	231	83	35.9%
(Hoa & Trang, 2017) [20]	Central	Binh Dinh	Pregnant women at Binh Dinh Provincial General Hospital from August 2015 to March 2016.	24–28 weeks of gestation	Pre-existing diabetes before pregnancy. Having conditions affecting glucose metabolism Using medications affecting glucose metabolism	IADPSG	Cross sectional	2015	2016	369	77	20.9%
(Hoa & Trang, 2018) [21]	South	District 2, Ho Chi Minh City	Pregnant women at District 2 Hospital, Ho Chi Minh City from October 2016 to April 2017	24–28 weeks of gestation	Inability to perform glucose tolerance tests. Assisted conception. Pre-existing diabetes before pregnancy. Having conditions affecting glucose metabolism	IADPSG	Cross sectional	2016	2017	264	50	18.9%
(Hong, 2022) [22]	North	Hanoi	Pregnant women at Hanoi Obstetrics and Gynaecology Hospital from August 2021 to May 2022	Age 18 years or above	Pre-existing diabetes before pregnancy Having conditions affecting glucose metabolism Using medications affecting glucose metabolism Cognitive impairments affecting the interview process. Currently undergoing inpatient treatment at the hospital Acute illnesses	IADPSG	Cross sectional	2021	2022	140	53	37.9%
(Hưng & Hùng, 2024) [23]	North	Hanoi	Pregnant women at Hanoi Obstetrics and Gynaecology Hospital from 1 January 2023, to 30 June 2023.	24–28 weeks of gestation	Pre-existing diabetes before pregnancy. Having conditions affecting glucose metabolism Using medications affecting glucose metabolism	IADPSG	Cross sectional	2023	2023	1450	423	29.17%
(Huong et al., 2023) [24]	Central	Hue	Pregnant women at the Obstetrics Department, Hue Central Hospital, from January 2021 to June 2022	24–28 weeks of gestation	Pre-existing diabetes before pregnancy. Having conditions affecting glucose metabolism Using medications affecting glucose metabolism Acute illnesses	IADPSG	Cross sectional	2021	2022	495	101	20.4%
(Le et al., 2025) [25]	North	Thai Binh	Pregnant women at Thai Binh Maternity Hospital and Kim Ngan Clinic in Thai Binh City from January to August 2023 and from January to February 2024	Age 18 years or above Pregnancy < 28 weeks Residing in Thai Binh Province	Pre-existing diabetes before pregnancy. Severe chronic disease.	IADPSG	Cross sectional	2023	2024	1106	300	27.1%
(Nga & Tra, 2019) [26]	South	Long An	Pregnant women at Long An General Hospital, from 2 January 2018, to 30 June 2018	20–27 weeks of gestation	Pre-existing diabetes before pregnancy. Having conditions affecting glucose metabolism Using medications affecting glucose metabolism	IADPSG	Cross sectional	2018	2018	404	62	15.35%
(Nga, 2023) [27]	South	Can Tho, An Giang, Soc Trang, Ca Mau (Mekong Delta Region)	Pregnant women in the Mekong Delta region: Can Tho Maternity Hospital, An Giang Obstetrics and Paediatrics Hospital, Soc Trang Obstetrics and Paediatrics Specialized Hospital, and Ca Mau Obstetrics and Paediatrics Hospital, from June 2017 to June 2022.	24–28 weeks of gestation Singleton pregnancy	Pre-existing diabetes before pregnancy Inability to perform OGTT or collect three blood samples. Conception through ovulation induction or in vitro fertilization. Having conditions affecting glucose metabolism Suffering from malignancies, severe medical conditions, cardiovascular diseases, or mental disorders.	IADPSG	Cross sectional	2017	2022	1727	295	17.10%
(Thuy et al., 2023) [28]	North	Hanoi	Pregnant women at National Hospital of Obstetrics and Gynaecology (Ha Noi) from January to March 2023.	Singleton pregnancy	Not mentioned	IADPSG	Cross sectional	2023	2023	574	251	43.73%
(Tam et al., 2022) [29]	South	Soc Trang	Pregnant women at Soc Trang Obstetrics and Paediatrics Specialized Hospital from November 2019 to November 2020.	24–28 weeks of gestation Singleton pregnancy	Having conditions affecting glucose metabolism Using medications affecting glucose metabolism Regular exposure to tobacco smoke or frequent alcohol consumption.	IADPSG	Cross sectional	2019	2020	1360	359	26.4%
(Tran et al., 2013) [30]	South	District 5, Ho Chi Minh City	Pregnant women at Hung Vuong Hospital, a tertiary referral maternity hospital in Ho Chi Minh City, from 1 December 2010, to 31 March 2011.	Age 18 years or above 24–32 weeks of gestation Singleton pregnancy Planning to give birth at the hospital.	Pre-existing diabetes before pregnancy. Referred from other hospitals or private clinics for managing prenatal complications or giving birth. Inability to complete the OGTT.	IADPSG	Cross sectional	2010	2011	2,772	565	20.4%
(Trang & Ha, 2013) [31]	North	Hanoi	Pregnant women at the National Hospital of Obstetrics and Gynaecology (Ha Noi) from May 2012 to August 2012	24–28 weeks of gestation	Not mentioned	IADPSG	Cross sectional	2012	2012	210	39	18.6%
(Tri et al., 2021) [32]	South	Ca Mau	Pregnant women at Ca Mau Obstetrics and Paediatrics Hospital from May 2020 to April 2021	Not mentioned	Pre-existing diabetes before pregnancy Having conditions affecting glucose metabolism Using medications affecting glucose metabolism Regular exposure to tobacco smoke or frequent alcohol consumption	IADPSG	Cross sectional	2020	2021	290	56	19%
(Tuan et al., 2023) [33]	South	Can Tho	Pregnant women at Phuong Chau International Hospital, Can Tho from August 2022 to March 2023	24–28 weeks of gestation	Pre-existing diabetes before pregnancy Having conditions affecting glucose metabolism Using medications affecting glucose metabolism	IADPSG	Cross sectional	2022	2023	300	99	33%
(Vi & Tuan, 2021) [34]	South	District 1, Ho Chi Minh City	Pregnant women at District 1 Hospital, Ho Chi Minh City, from October 2019 to May 2020	Age 18 years or above 24–28 weeks of gestation	Pre-existing diabetes before pregnancy Having conditions affecting glucose metabolism	IADPSG	Cross sectional	2019	2020	390	128	32.8%

Note: GDM Gestational Diabetes Mellitus, IADPSG International Association of Diabetes and Pregnancy Study Groups, OGTT Oral Glucose Tolerance Test.

**Table 4.** Summary of risk factors for gestational diabetes mellitus in Vietnam.

Authors, year	Study design	Total participants	Participants characteristics	Risk factors
(Anh et al., 2024) [35]	Case-control	68	33 women with GDM, 35 women without GDM	History of GDM: Increases the risk of GDM in the current pregnancy (OR = 7.1). Previous macrosomia (infant ≥ 4000 g): Increases the risk of GDM (OR = 6.3). History of stillbirth: (OR = 5.0).
(Do & Nguyen, 2018) [16]	Prospective cohort	80	40 gestational diabetes mellitus and 40 non-gestational diabetes mellitus	Average maternal age: The GDM group had a significantly higher average age (31.38 ± 5.17 years) compared to the non-GDM group (27.70 ± 5.56 years). Pre-pregnancy BMI: A higher percentage of women with a BMI ≥23 kg/m^2^ (overweight or obese) was found in the GDM group (40%) compared to the non-GDM group (12.5%). Family History: The proportion of women with a family history of diabetes (first-degree relatives) was higher in the GDM group (27.5%) than in the non-GDM group (7.5%). Previous Macrosomia: The rate of women with a history of giving birth to infants weighing ≥3500 g was higher in the GDM group (27.5%) compared to the non-GDM group (10%).
(Duyen & Thuong, 2019) [17]	Cross-sectional	260	Women with gestational age between 24 and 28 weeks	Maternal age ≥ 35 years (PR = 3.51). Previous macrosomia ≥ 4000 g (PR = 4.64) Pre-pregnancy BMI ≥ 25 kg/m^2^ (PR = 2.08). Family history of diabetes (PR = 3.16)
(Hang et al., 2016) [18]	Cross-sectional	465	Women with gestational age between 24 and 28 weeks	Family history of diabetes (first-degree relatives: parents, siblings) (OR = 2.8). History of stillbirth (OR = 1.04)
(Hien et al., 2025) [19]	Cross-sectional	231	Women with gestational age between 24 and 28 weeks	Maternal age ≥ 35 years: The prevalence of GDM in this group is 50%, higher than the <35 years group (31.4%). History of vaginitis: 100% of pregnant women with a history of vaginitis have GDM. Family history of diabetes (first-degree relatives): 100% of pregnant women with a family history of diabetes have GDM. Fertility treatment: 100% of pregnant women conceived through assisted reproductive technology have GDM. Weight gain ≥7 kg by the end of the second trimester: The prevalence of GDM in this group is 50.0%, higher than those with weight gain <7 kg (30.1%). Foetal weight ≥90th percentile: The prevalence of GDM in this group is 46.2%, higher than those with foetal weight <90th percentile (29.3%). Polyhydramnios: The prevalence of GDM in women with polyhydramnios is 78.6%, higher than those without polyhydramnios (33.2%). Positive urine glucose test (Glucosuria): 100% of pregnant women with positive urine glucose tests have GDM.
(Hoa & Trang, 2017) [20]	Cross-sectional	369	Women with gestational age between 24 and 28 weeks	Family history of diabetes (first-degree relatives) (OR = 2.0). History of stillbirth (OR = 15.1). Pregnant women with ≥2 risk factors (OR = 7.5)
(Hoa & Trang, 2018) [21]	Cross-sectional	264	Women with gestational age between 24 and 28 weeks	Family history of diabetes (first-degree relatives) (OR = 5.6). Glucosuria (OR = 17.3)
(Hong, 2022) [22]	Cross-sectional	140	Women with gestational age between 24 and 28 weeks	Maternal age 30–34 years (OR = 4.7). Maternal age ≥ 35 years (OR = 3.1). Family history of diabetes (first-degree relatives) (OR = 4.3). Parity ≥3 pregnancies (OR = 4.0). Pre-pregnancy BMI ≥23 kg/m^2^ (OR = 1.4)
(Hưng & Hùng, 2024) [23]	Cross-sectional	1450	Women with gestational age between 24 and 28 weeks	Maternal age ≥35 years has a higher risk of GDM compared to those <35 years. The prevalence of GDM was 15.13% among women with a pre-pregnancy BMI ≥23 kg/m^2^, compared to 7.5% among those with a BMI <23 kg/m^2^
(Huong et al., 2023) [24]	Cross-sectional	495	Women with gestational age between 24 and 28 weeks	Maternal age ≥35 years (OR = 2.74) Pre-pregnancy BMI ≥ 23 kg/m^2^ (OR = 2.35) Previous Macrosomia ≥ 3500 g (OR = 3.39) Family history of diabetes (OR = 2.44) History of stillbirth (OR = 1.81)
(Le et al., 2025) [25]	Cross-sectional	1106	Women with gestational age between 24 and 28 weeks	Maternal age 25–34 years (OR = 2.0). Maternal age ≥ 35 years (OR = 3.0). Pre-pregnancy BMI ≥ 23 kg/m^2^ (OR = 1.6) Family history of diabetes (OR = 1.9). Fertility treatment (OR = 2.3). History of GDM (OR = 3.1)
(Nga & Tra, 2019) [26]	Cross-sectional	404	Women with gestational age between 24 and 28 weeks	Previous Macrosomia ≥ 4000 g (OR = 3.37) Positive urine glucose test (glucosuria) (OR = 3.06) High-carbohydrate diet (OR = 5.72)
(Nga, 2023) [27]	Cross-sectional	1727	Women with gestational age between 24 and 28 weeks	Maternal age ≥ 25 years (OR = 1.72). Kinh ethnicity (OR = 1.81). Urban residence (OR = 1.34). Education level below high school (OR = 1.46). Family history of diabetes (OR = 1.68). Pre-pregnancy BMI ≥ 25 kg/m^2^ (OR = 2.79) Weight gain ≥ 12 kg (OR = 1.50). History of miscarriage (OR = 1.36). Previous birth of infant ≥ 4000 g (OR = 4.08). History of GDM (OR = 5.66).
[36]	Cross-sectional	106	Women with gestational age between 24 and 28 weeks	Age ≥ 25: The prevalence of GDM is 49.1%. The prevalence of GDM increases with age, peaking at 50.0% in the 30–34 age group. Pre-pregnancy BMI ≥23 kg/m^2^: The prevalence of GDM among overweight and obese women (20.0%) is significantly higher compared to those who are not overweight or obese (2.1%). Family history of diabetes (first-degree relatives): The prevalence is 14.2%. A family history of diabetes increases the prevalence of GDM (60.0% compared to 40.0% in those without a family history). History of abnormal obstetric outcomes (stillbirth, preterm birth): The prevalence is 17.9%. A history of abnormal obstetric outcomes increases the prevalence of GDM (60.0% compared to 40.0% in those without such history)
(Nguyen et al., 2018) [37]	Prospective cohort	1987	Women with gestational age between 24 and 28 weeks	Higher physical activity levels during pregnancy are associated with a lower risk of GDM. Moderate-intensity activity and caregiving activities during pregnancy are linked to reduced GDM risk. No significant associations were found between GDM and sedentary time, light-intensity activity, vigorous-intensity activity, occupational activity, sports/exercise, transportation, or meeting exercise guidelines. Compared to women without GDM, those with GDM are older, have higher BMI, blood pressure, and a stronger family history of diabetes. They also have lower overall physical activity levels, moderate-intensity activity, and caregiving activity.
(Nhu et al., 2017) [38]	Cross-sectional	134	Women undergoing in vitro fertilization	Physical exercise before and during pregnancy: Reduces the risk of GDM. The prevalence of GDM is 12.7% in those who exercise compared to 39.7% in those who do not. Exercise before and during pregnancy decreases the risk of GDM by 58% (PR = 0.42)
(Pham et al., 2024) [39]	Case-control	210	114 women with GDM, 96 women without GDM	No significant difference in Methylenetetrahydrofolate reductase C677T gene polymorphism (CC, CT, and TT) between the two groups. Weight, glucose levels at 0, 1, and 2 hours during OGTT, and folic acid levels were significantly higher in the GDM group compared to the non-GDM group.
(Phuong & Phung, 2011) [40]	Cross-sectional	761	Women with gestational age between 13 and 28 weeks	Family history of diabetes (OR = 6.37). History of stillbirth (OR = 1.13).
(T. T. Nguyen et al., 2020) [41]	Cross-sectional	706	Women with gestational age between 24 and 28 weeks	Maternal age > 30 years (OR = 2.38). History of GDM (OR = 12.21). Pre-pregnancy BMI ≥ 23 kg/m^2^ (OR = 10.78). Previous macrosomia > 3800 g (OR = 4.66)
(Tam, 2017) [42]	Cross-sectional	1511	Women with gestational age between 13 and 28 weeks	Maternal age ≥ 35 years (OR = 4.0). Parity: Second pregnancy (OR = 1.5), Third or more pregnancies (OR = 2.2). Pre-pregnancy BMI: Overweight (BMI 23 - <25 kg/m^2^) (OR = 4.5), Obesity (BMI ≥ 25 kg/m^2^) (OR = 11.2). Obstetric history: Stillbirth (OR = 2.3), Miscarriage (OR = 2.4), Previous macrosomia (OR = 3.1). Family history of diabetes (first-degree relatives) (OR = 2.5). Chronic hypertension history: (OR = 2.7). Dietary habits: Consumption of animal fat (OR = 1.5); Drinking soft drinks ≥5 days/week, ≥1 cup/day (OR = 4.8); Eating yogurt ≥5 days/week, ≥1 box/day reduces the risk of GDM. Combined factors: Overweight/obesity with consuming ≥3 cans of soft drinks/≥3days/week (OR = 11.7). Work nature: More sitting time than walking (OR = 2.7). Number of risk factors: Having ≥3 risk factors (OR = 12.1)
(Trang & Ha, 2013) [31]	Cross-sectional	210	Women with gestational age between 24 and 28 weeks	Advanced maternal age: The prevalence of GDM increases with age, from the lowest in the ≤24 years group (2.6%) to the highest in the ≥40 years group (50%). Pre-pregnancy BMI ≥23 kg/m^2^: The prevalence of GDM is higher in the BMI ≥23 kg/m^2^ group (40%) compared to the BMI <18.5 kg/m^2^ group (14.6%) and the BMI 18.5–22.9 kg/m^2^ group (15.1%). High parity (≥3 pregnancies): The prevalence of GDM is higher in women with ≥3 pregnancies (28.0%) compared to those with 1–2 pregnancies (13.3%).
(Trang et al., 2020) [43]	Cross-sectional	120	Pregnant women diagnosed with GDM	Advanced maternal age: The highest prevalence of GDM is observed in the ≥35 years group (33.3%) and the lowest in the <25 years group (5%). Pregnancy history and parity: The average number of pregnancies among participants is 2.6 ± 1.1. A total of 34 women had ≥4 pregnancies, representing the highest proportion (28.3%)
(Trang et al., 2024) [44]	Cross-sectional	350	Women with gestational age between 24 and 28 weeks	Maternal age ≥ 25 years (OR = 3.1). Family history of diabetes (OR = 7.07). Education level of high school or above (OR = 3.86). Consumption of sweetened milk (OR = 59.15).
(Tuan et al., 2023) [33]	Cross-sectional	300	Women with gestational age between 24 and 28 weeks	Maternal age 30–34 years (Univariate analysis: OR = 2.1) (Multivariate analysis: OR = 2.4) Age ≥ 35 years (Univariate analysis: OR = 2.5) (Multivariate analysis: OR = 3.0) Pre-pregnancy BMI 23–24.9 kg/m^2^ (Univariate analysis: OR = 2.0) Multivariate analysis: OR = 2.1) Primiparous women: OR = 2.0 multivariate analysis.
(Van et al., 2017) [45]	Cross-sectional	78	Women undergoing in vitro fertilization	Pre-pregnancy BMI: Higher in the GDM group compared to the non-GDM group (22.8 ± 3.5 vs. 21.1 ± 3.1 kg/m^2^). Pre-pregnancy BMI ≥ 23 kg/m^2^ (OR = 2.43). Glucosuria (OR = 5.67)
(Vi & Tuan, 2021) [34]	Cross-sectional	390	Women with gestational age between 24 and 28 weeks	History of GDM (PR = 2.01). Pre-pregnancy BMI ≥ 23 kg/m^2^ (PR = 1.96). Education beyond high school (college, university, postgraduate) (PR = 2.0)
(Yen et al., 2018) [46]	Retrospective cohort	217	Women undergoing in vitro fertilization	Maternal age is associated with an increased risk of GDM (OR = 1.02). Pre-pregnancy BMI is associated with an increased risk of GDM (OR = 1.03)

Note: BMI Body Mass Index, GDM Gestational Diabetes Mellitus, OR Odds Ratio, PR Prevalence ratio.

### Data analysis

Data were synthesised descriptively across prevalence, risk factors, outcomes, knowledge, and interventions, with stratification by study and population characteristics where appropriate. Owing to substantial heterogeneity, no meta-analysis was conducted; findings were presented narratively and in tables. The review was reported in accordance with the Preferred Reporting Items for Systematic Reviews and Meta-Analyses Extension for Scoping Reviews (PRISMA-ScR) guidelines [47].

To provide a descriptive indication of the GDM burden represented in the available Vietnamese evidence, a sample-size-weighted average prevalence was calculated for a subset of hospital-based studies that used International Association of Diabetes and Pregnancy Study Groups (IADPSG)-based diagnostic criteria (Table 3) and had broadly comparable populations and eligibility criteria. This average was calculated as the sum of each study prevalence multiplied by its sample size, divided by the total sample size across the eligible studies [48]. Study sample size was used as the weighting factor; however, no meta-analytic methods were applied, as the estimates were intended to provide descriptive ranges consistent with the scoping objective of evidence mapping.

## Results

### Characteristics of the selected studies

A comprehensive literature search identified records from both international (*n* = 275) and local databases (*n* = 124), as detailed in Figure 1. Ultimately, 76 studies met the inclusion criteria and were included in this scoping review:

- One study (1.3%) examined prevalence, risk factors, and maternal/neonatal outcomes
- 18 studies (23.7%) addressed both prevalence and risk factors
- 5 studies (6.6%) examined prevalence and outcomes
- 9 studies (11.8%) focused exclusively on risk determinants
- 13 studies (17.1%) reported prevalence across different populations and regions.
- 6 studies (7.9%) assessed knowledge of GDM, including symptoms, diagnosis, and prevention.
- 17 studies (22.4%) investigated maternal and neonatal outcomes
- 7 studies (9.2%) evaluated dietary, lifestyle, or digital interventions for glycaemic management.

### Prevalence of GDM in Vietnamese populations

#### Prevalence of GDM among Vietnamese migrant populations

Among the 37 studies reporting GDM prevalence, ten studies (27.0%) were conducted among Vietnamese migrants in high-income countries [5–10,12–15]. Six studies compared GDM prevalence among Vietnamese women with that of other ethnic groups or the general population in the host country (Table 1), while four examined differences according to maternal country of birth (Table 2). Substantial heterogeneity was observed in data sources and diagnostic approaches, with many studies relying on secondary data without specified diagnostic criteria, while others applied oral glucose intolerance test (OGTT) based protocols with varying thresholds.

Despite this variability, evidence consistently showed a higher prevalence of GDM among Vietnamese women compared with other ethnic groups and the general population in host countries (Table 1). Maternal country of birth emerged as a strong determinant, with Vietnam-born women exhibiting higher GDM risk than their native-born counterparts (Table 2). These findings were interpreted descriptively because differences in study periods, and diagnostic practices limited direct quantitative comparison.

#### Prevalence of GDM among local Vietnamese pregnant women

The remaining 27 prevalence-reporting studies were conducted in Vietnam; of these, only one community-based study using IADPSG criteria reported a GDM prevalence of 20.38% [42]. The other 26 studies were hospital-based.

Four did not apply IADPSG criteria and instead used risk-based screening with fasting glucose [49] or 50 g glucose challenge tests with varying thresholds [40], while others applied different cut-offs for the 75 g OGTT [36,50]. Accordingly, these studies were reported separately and not included in the descriptive aggregation.

Of the 23 studies applying IADPSG criteria, two focused exclusively on pregnancies conceived through in vitro fertilisation and reported prevalence estimates of 55.1% in Hanoi [45] and 25.4% in Ho Chi Minh City [38]. Two others excluded women with previous GDM and reported lower estimates (10.2% and 8.8%) [51,52], likely reflecting underestimation. These four studies were retained in the evidence map but excluded from the descriptive aggregation because of their selected populations or eligibility criteria.

The remaining 19 IADPSG-based studies (*n* = 13,382) with comparable populations and eligibility criteria (Table 3) had a sample -size-weighted average prevalence of 23.24% (2010–2024), indicating a substantial GDM burden among Vietnamese women.

#### Temporal variation in reported GDM prevalence in Vietnam (2010–2024)

Aggregated prevalence estimates from eligible IADPSG-based studies (Table 3) showed substantial fluctuations between 2010 and 2024, with studies conducted in later periods generally reporting higher prevalence estimates than those conducted earlier (Figure 2). The reported rates declined from 20.4% (2010–2011) to 13.6% (2017–2018), then rose to 27.8% (2019–2020), peaked at 33.3% (2022–2023), and stabilised at 28.7% (2023–2024), indicating an overall rising burden in recent years.

**Figure 2.**
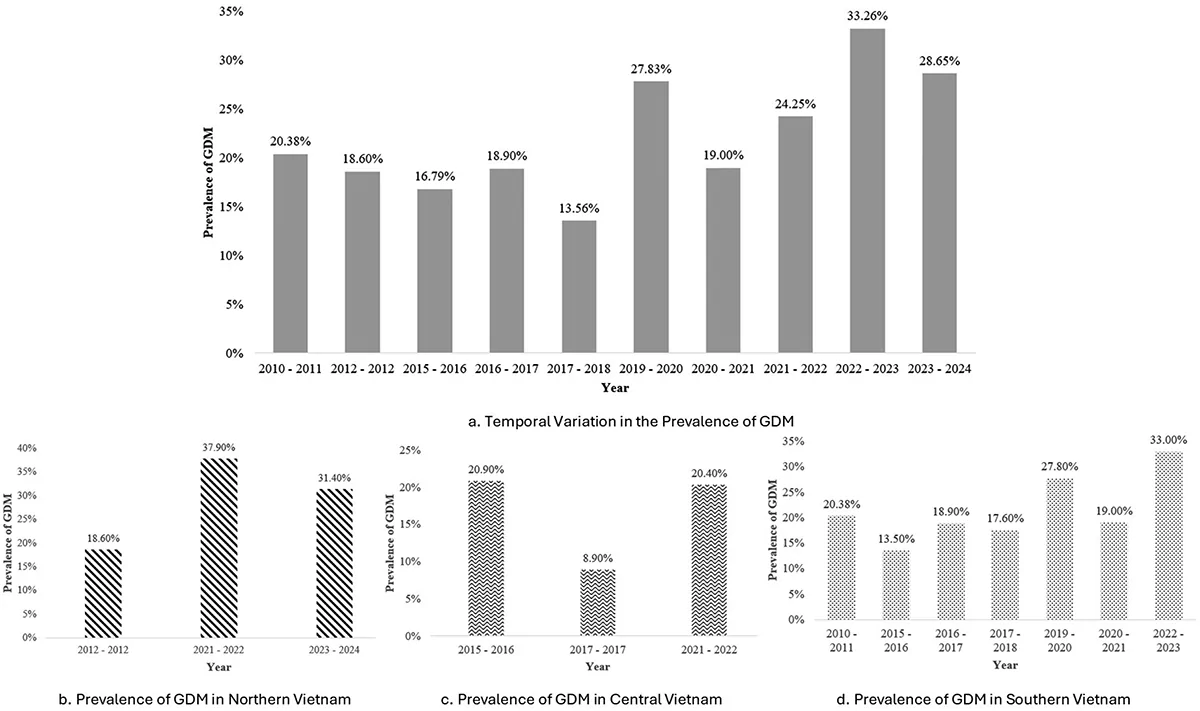
Reported prevalence of gestational diabetes mellitus (GDM) in Vietnam by study period and region, 2010–2024.

Regional analyses revealed notable geographic disparities (Figure 2). Northern Vietnam showed higher prevalence estimates in more recent study periods, Central Vietnam showed a later but emerging increase, and Southern Vietnam demonstrated fluctuations with higher estimates in some recent periods, suggesting regional differences in population characteristics, urbanisation, and healthcare practices.

#### Regional distribution of GDM prevalence in Vietnam

Regional analysis showed clear disparities in aggregated GDM prevalence across Vietnam (Table 3). Central Vietnam reported the lowest mean prevalence (15.91%; range 8.9–20.9%) [16,20,24]. Northern Vietnam had the highest regional mean (30.96%), with the highest single estimate reported in Hanoi (43.73%) [28]. Southern Vietnam showed intermediate prevalence (mean 21.1%) but substantial variability, ranging from 15.35% in Long An [26] to 33.0% in Can Tho [33]. Within Ho Chi Minh City, the aggregated GDM prevalence averaged 20.71%, with the highest estimate reported in the central urban district (32.8%) [34].

### Risk factors for GDM in Vietnamese women

#### Demographic risk factors

Among 27 studies examining risk factors, maternal age was the most consistent demographic determinant of GDM (Table 4). Women aged ≥ 35 years had a substantially higher risk, with adjusted odds ratios ranging from 2.5 to 4.0 compared with younger women [17,22,25,33,42], and a higher proportion of GDM cases (50% vs 31.4%, *p* = 0.022) [19]. Women aged 30–34 years also had an increased risk (OR ≈ 2.0–2.4) [25,33], and up to 4.7-fold higher risk compared with women < 25 years [22]. Prevalence increased progressively with age, from 2.6% in women ≤ 24 years to 50% in women ≥ 40 years [31].

#### Clinical risk factors

Elevated pre-pregnancy BMI was a major risk factor for GDM. Women with BMI ≥ 23 kg/m^2^ had higher GDM prevalence (15–40%) [16,23,31,36] and increased risk, with reported odds ratios ranging from 1.4 to 11.2 compared with women of normal BMI [22,24,25,33,41]. Risk increased progressively with higher BMI, with overweight women (BMI 23 – < 25 kg/m^2^) having a 4.5-fold higher risk and obese women (BMI ≥ 25 kg/m^2^) an 11.2-fold higher risk of GDM [42].

Obstetric history was an important risk factor for GDM. Previous macrosomia (≥ 3500 g) was strongly associated with GDM (OR = 3.37–6.3) [17,24,26,35,41,42]. A history of stillbirth was also associated with increased risk (OR = 1.04–15.1) [18,20,24,35,40,42], while previous miscarriage was associated with moderately increased risk (OR = 1.36–2.4) [27,42].

Family and personal history of diabetes were strong predictors of GDM. A family history of diabetes, particularly in first-degree relatives, was associated with increased risk (OR = 1.68–7.07) [18,20–22,24,25,27,40,42,44], while a prior history of GDM was associated with a substantially higher recurrence risk (OR = 2.01–12.21) [25,27,34,35,41].

Several current pregnancy conditions were associated with increased GDM risk. Glucosuria was a consistent predictor (OR = 3.06–17.3) [19,21,26,45]. Parity showed mixed associations, but higher parity was linked to increased risk in some studies (≥ 3 pregnancies: 28% vs 13.3%; OR = 4.0) [22], with risk increasing by pregnancy order (OR = 1.5 for second pregnancy; OR = 2.2 for third or more) [42]. Polyhydramnios was associated with markedly higher GDM prevalence (78.6% vs 33.2%) [19], and chronic hypertension increased risk (OR = 2.7) [42].

#### Dietary risk factors

A high-carbohydrate diet significantly increased risk (OR = 5.72) [26], as did frequent consumption of soft drinks (OR = 4.8) and animal fat (OR = 1.5) [42]. The combined effect of overweight/obesity and frequent soft drink intake further increased risk (OR = 11.7) [42]. Sweetened milk consumption was associated with particularly high risk (OR = 58.15) [44], whereas frequent yogurt intake (≥5 days/week) was associated with reduced risk [42].

#### Lifestyle related risk factors

Women who exercised had a lower GDM prevalence (12.7% vs 39.7%), corresponding to a 58% risk reduction [38]. Higher levels of moderate-intensity activity were protective [37], whereas sedentary behaviour increased risk (OR = 2.7) [42].

#### Genetic risk factors

Evidence on genetic risk factors for GDM in Vietnamese women remains limited. One study on the *MTHFR* C677T polymorphism found no significant association with GDM, likely due to small sample size [39]. In contrast, a recent multi-omics study (*n* = 1,086) identified five loci (*HSD11B1, NEK7, COMMD10, KLRC4*, and *OCEL1*) associated with GDM, supporting a polygenic model in which the cumulative effect of multiple variants improves risk prediction compared with single-gene analyses [53].

### Current knowledge on gestational diabetes mellitus in Vietnam

#### General knowledge of GDM

Evidence indicates that knowledge of GDM among Vietnamese pregnant women remains limited. Quantitative studies reported that only 51.7–60% of women correctly identified GDM, while a small proportion had no knowledge of the condition [54,55]. Qualitative studies revealed misconceptions about causes, testing, and health implications, with many women experiencing confusion and anxiety at diagnosis [56–58]. Family members frequently downplayed the seriousness of GDM, perceiving it as temporary, which sometimes left women feeling unsupported despite medical advice [58]. Inadequate counselling was commonly reported, with advice often limited to reducing sugar intake without comprehensive dietary or lifestyle guidance. Structural barriers, including cost and access to specialist care, further limited education and support [56,58].

#### Knowledge of risk factors about GDM

While 70.3% recognised obesity and 60.3% identified previous GDM as risk factors, awareness of other key determinants, such as advanced maternal age and family history of diabetes, was lower [54]. Only 35.3% of women demonstrated adequate knowledge of GDM prevention, and higher education was associated with better knowledge (OR = 2.94) [59]. Qualitative evidence also revealed persistent misconceptions, with many pregnant women uncertain about true GDM risk factors and often misattributing unrelated symptoms to the condition [56].

#### Knowledge of symptoms, diagnosis, complications, management and prevention of GDM

Three quantitative studies showed that knowledge of GDM symptoms and diagnosis among Vietnamese pregnant women was variable. While 73.1% recognised common symptoms and 70.6% were aware of the recommended screening period (24–28 weeks), only 23.3% correctly identified diagnostic fasting glucose levels, and 72.8% were unaware of diagnostic thresholds [54]. Awareness of complications and prevention was high, with over 98% recognising maternal and foetal risks and 99.7% believing GDM could be prevented [54]; however, only 30.1% demonstrated appropriate preventive behaviours [59]. Knowledge also appeared higher among women aged over 35 years, of whom 76.9% met the predefined threshold for adequate knowledge [55].

Three qualitative studies identified dietary management as a major concern. Before screening, many women had a limited understanding of why testing was required, and a positive result was often followed by disbelief, anxiety, guilt, and uncertainty [56–58]. Women commonly attributed GDM to rapid gestational weight gain, cravings for rice, or consumption of sweets and starchy foods, and some linked abnormal results to a recent meal, late-night eating, or specific foods such as jackfruit [57]. Dietary advice was frequently limited to ‘reduce starch,’ often interpreted as reducing rice, a staple food in Vietnam. Without appropriate guidance on food substitutions, some women adopted overly restrictive diets, leading to hunger and uncertainty about nutritional adequacy [56].

### Outcomes associated with gestational diabetes mellitus in Vietnam

#### Maternal outcomes

Caesarean Birth: GDM was associated with a higher risk of caesarean birth, with reported rates ranging from 41.2% to 81.2% among women with GDM, substantially higher than in non-GDM pregnancies [29,42,52,60–66]. Comparative studies reported caesarean rates of 44.2% vs 36.5% (*p* = 0.007) [62] and 71.4% vs 55.9% (*p* = 0.003), with GDM increasing caesarean risk by 1.7-fold [64]. Common indications included previous caesarean scar, macrosomia, and foetal distress [29,42,52,60–66].

Preterm Birth: GDM was associated with an increased risk of preterm birth, with reported rates ranging from 9.1% to 33.6% in Vietnam [29,42,50,52,60,61,63,67]. Women with GDM had a 40% higher risk of preterm birth (OR = 1.40) [68] and a higher likelihood of early labour induction (OR = 1.51) [69]. The risk was further increased when GDM coexisted with obesity (BMI ≥ 27.5 kg/m^2^), with a combined OR of 2.42 [68].

Psychological Outcomes: Depressive symptoms were relatively common among women with GDM, with a reported prevalence of 16.82%, higher than the 13.5% previously reported in the general pregnant population [70]. Lower levels of social support, particularly from family and friends, were associated with higher odds of depression, with a stronger effect observed among women with GDM than those without [71].

Breastfeeding Outcomes: Breastfeeding duration tended to be shorter among women with GDM, partly due to higher rates of preterm birth and neonatal intensive care admission, which delayed breastfeeding initiation. Maternal insulin resistance may also impair lactogenesis II, delaying milk production and contributing to earlier breastfeeding cessation [72].

Group B Streptococcus Colonisation: GDM was associated with increased susceptibility to Group B Streptococcus (GBS) colonisation, with prevalence of 34.7% in women with GDM compared with 16.3% in non-GDM pregnancies. GDM increased the risk of GBS colonisation 5.3-fold (OR = 5.3; 95% CI: 3.4–8.3), and co-occurrence of GDM and GBS was associated with higher risks of preterm birth (OR = 4.9), caesarean birth (OR = 4.1), and premature rupture of membranes (OR = 2.3) [64].

Long-term Maternal Outcomes: Women with GDM were at high risk of persistent glucose abnormalities and progression to type 2 diabetes postpartum. At 6–12 weeks postpartum, 25.9% had abnormal glucose metabolism (2.7% diabetes; 22.2% impaired glucose tolerance) [73]. Other studies reported 11.8% developing type 2 diabetes and up to 47.3% prediabetes within six weeks postpartum [74], while another reported 42.2% prediabetes and 1.5% diabetes [75].

#### Neonatal outcomes

Macrosomia (≥ 4000 g) was one of the most consistent neonatal outcomes associated with GDM, with reported prevalence ranging from 2.1% to 15.8% among infants of mothers with GDM [29,32,51,60,61,63,65]. Infants of GDM mothers were significantly heavier, with one study reporting a mean birth weight 170 g higher than in non-GDM pregnancies (*p* = 0.046) [76]. Among macrosomic births, 73% of mothers had GDM, and 87.6% of these had poor glycaemic control [77].

Infants of mothers with GDM frequently required specialised neonatal care, with admission rates ranging from 23.7% to 24.2%. The main reasons for admission included prematurity (47.9%), respiratory distress (38.4%–48.0%), and monitoring for hypoglycaemia in macrosomic infants (13.7%) [29,65].

#### Outcomes among Vietnamese diaspora populations

Only two studies reported GDM-related outcomes among Vietnamese-origin women. Vietnam-born women with GDM had higher rates of pre-eclampsia (6.9%) and polyhydramnios (12.5%) than those with normal glucose tolerance; 25% developed permanent diabetes within nine years, compared with 9% of Australian-born women with GDM over 12 years [12]. Pre-eclampsia was also more common among Vietnamese-born than Australian-born women with GDM (7.5% vs 5.3%) [14].

#### Intervention studies on gestational diabetes mellitus in Vietnam

Seven quasi-experimental studies (pre–post design without control groups) evaluated interventions for women with GDM in Vietnam, with six conducted at major maternity hospitals in Ho Chi Minh City, none involved Vietnamese diaspora populations. Interventions included dietary and lifestyle modification (4 studies) [78–81], dietary intervention alone (2 studies) [82,83], and dietary intervention combined with digital nutrition counselling (1 study) [84].

Dietary interventions showed substantial improvements in glycaemic control, with 71.2–87.2% achieving target glucose levels within 3–7 days under carbohydrate-restricted or low-glycaemic-index diets [82], and up to 96% achieving glycaemic stabilisation after 3 weeks with structured macronutrient-balanced diets [83].

Combined dietary and lifestyle interventions in Vietnam, including structured diets and ≥30 minutes/day of moderate physical activity, achieved a mean glycaemic control, ranging from 59.2% to 87.2% [78–81]. Glycaemic stabilisation improved with longer intervention duration (e.g. 71.2% at day 3 to 84.8% at day 5; up to 87.2% at day 7) [80]. Intervention success was higher in women <35 years (3.2 times more likely to achieve control) and those with pre-pregnancy BMI <25 kg/m^2^ (10.84 times more likely) [78].

Dietary plus digital counselling resulted in 80% of participants achieving glycaemic and weight control targets after two weeks, with a 3.1 mmol/L reduction in 2-hour postprandial glucose [84].

Management following unsuccessful lifestyle intervention was inconsistently reported, and three studies did not describe treatment escalation [79,83,84]. Among studies reporting pharmacotherapy escalation, 5.8–17.4% of participants required consideration of, or were prescribed, insulin therapy [78,80–82].

## Discussion

### GDM burden in Vietnam in a regional and global context

The sample-size-weighted average prevalence of GDM from comparable Vietnamese studies was 23.24% between 2010 and 2024. Although this figure represents a restricted aggregate of selected hospital-based studies rather than a nationally representative prevalence estimate, its magnitude suggests a substantial GDM burden within the populations studied. This estimate is higher than the global average of 14.2% and the Southeast Asian estimate of 20.8% [85]. Within Southeast Asia, Vietnam’s estimate appears lower than Thailand but higher than estimates reported for Singapore (20.0%) [86] and Malaysia (21.5%) [87].

Reported GDM prevalence in Vietnam appeared to increase over time, while diaspora studies generally found a higher burden among Vietnamese women than host populations. These patterns suggested that risk was shaped by biological, sociocultural, dietary, migration-related, socioeconomic, and health-system factors, underscoring the need for culturally responsive care in both domestic and migrant settings.

### From candidate risk factors to risk-stratified antenatal care

The risk factors identified in Vietnamese studies are broadly consistent with international evidence. Advanced maternal age was one of the most consistent determinants of GDM, aligning with meta-analytic evidence showing a dose–response increase in GDM risk with increasing by 7.9% per year of maternal age [88], and age ≥35 years also predicting GDM recurrence [89]. Elevated pre-pregnancy BMI was another important and consistent candidate risk factor. International evidence similarly shows higher GDM risk among women with obesity, especially when combined with advanced maternal age, and highlights excessive gestational weight gain as an additional contributor [90,91]. Family history of diabetes, prior GDM, adverse obstetric history, and hypertensive disorders were also frequently reported in Vietnamese studies and are consistent with broader evidence [92,93]. These findings support the need to move from general GDM awareness towards risk-stratified antenatal care.

Diet and physical activity are especially important because they are modifiable and directly relevant to implementation. Vietnamese studies reported associations between GDM and high-carbohydrate diets, frequent soft drink consumption, animal fat intake, sweetened milk consumption, and physical inactivity. However, the very large estimate for sweetened milk consumption (adjusted OR = 58.15; 95% CI: 12.12–278.9) came from a single-centre cross-sectional study using convenience sampling and was highly imprecise [44]. It should therefore be interpreted cautiously as a context-specific association rather than a generalisable effect estimate.

International evidence suggests that diet quality may be more important than carbohydrate restriction alone. High-quality dietary patterns are associated with lower GDM risk, whereas low-carbohydrate diets high in animal protein and saturated fat may increase risk [94]. Higher glycaemic load is associated with increased GDM risk, while dietary fibre appears protective [95]. Similarly, physical activity before and during pregnancy is associated with lower GDM risk, whereas sedentary behaviour increases risk [96,97]. These findings indicate that counselling should avoid simplistic messages such as ‘reduce starch’ and instead promote culturally appropriate dietary quality, fibre-rich foods, healthy carbohydrate choices, portion control, and feasible physical activity.

Several potentially important determinants remain underexplored in Vietnam. Sleep quality and abnormal sleep duration have been associated with GDM risk internationally [98], but evidence from Vietnamese pregnant women is lacking. Similarly, no Vietnamese studies have yet examined the gut microbiome in relation to GDM, despite growing international evidence linking GDM with microbial dysbiosis, altered bile acid and branched-chain amino acid metabolism, inflammation, and gut barrier dysfunction [99–103]. Probiotic supplementation has also been associated with reduced GDM risk in meta-analyses, particularly when initiated before 20 weeks of gestation [104]. These emerging areas should be considered hypothesis-generating rather than practice-changing at this stage, but they point to future opportunities for locally relevant research integrating diet, microbiome, metabolism, and maternal glycaemic health.

### Knowledge, counselling, and culturally appropriate self-management

Knowledge of GDM among Vietnamese pregnant women remained incomplete, consistent with findings from other Asian settings [105,106]. Although women often recognised links with diet and sugar intake, they lacked practical guidance on meals, physical activity, and family-supported self-management. In a rice-based dietary context, generic advice to ‘reduce starch’ could lead to excessive restriction, hunger, anxiety, and poor adherence. Education should therefore be culturally specific, practical, and family-inclusive, with clear guidance on portions, staple-food modification, healthier beverages, and safe physical activity.

### Maternal, neonatal, and intergenerational implications

Maternal and neonatal outcomes reported in Vietnamese studies were consistent with international evidence, with GDM associated with higher risks of caesarean birth, preterm birth, macrosomia, neonatal care admission, hypertensive disorders, and preeclampsia [107]. However, caesarean birth rates varied considerably across settings. The highest rate, 81.2% among 308 women with GDM, was reported in a single hospital-based study and was attributed entirely to obstetric indications rather than uncontrolled glycaemia. The most common indication was pain related to a previous caesarean scar (39.0%), followed by fetal distress, arrested labour, and pre-eclampsia [29]. This estimate should therefore be interpreted as context-specific and not as an effect of GDM alone.

This review also highlights less frequently discussed outcomes, including depressive symptoms, shorter breastfeeding duration, Group B Streptococcus colonisation, and postpartum glucose abnormalities. International evidence supports increased risks of depression, urinary tract infection, and Group B Streptococcus colonisation among women with GDM [108–110]. These findings suggest that GDM care should be integrated with broader maternal health services, including mental health support, breastfeeding support, infection risk management, and postpartum metabolic monitoring.

Long-term follow-up is a particularly important implementation gap. International evidence shows that women with prior GDM are at substantially increased risk of type 2 diabetes, with especially high risk reported in South and Southeast Asian populations [111]. Offspring exposed to GDM also have higher risks of adiposity, elevated BMI, blood pressure, dyslipidaemia, and insulin resistance [112]. In Vietnam, postpartum follow-up evidence remains limited, and routine postnatal glucose testing may be inconsistently implemented. Strengthening postpartum care after GDM could therefore serve as an entry point for long-term non-communicable disease prevention for both mothers and children.

### Intervention evidence and the need for implementation-oriented trials

Vietnamese intervention studies suggest that dietary and lifestyle interventions can improve short-term glycaemic control among women with GDM. These findings are consistent with international evidence showing that diet-only and combined diet and physical activity interventions can reduce GDM incidence and improve glycaemic outcomes [113]. Specific dietary approaches, including DASH, low-glycaemic-index, and Mediterranean-style diets, have been associated with improved fasting and postprandial glucose, insulin sensitivity, and reduced insulin therapy requirements [114–116]. Nutritional supplements such as myo-inositol, probiotics, fish oil, and micronutrients have also shown potential benefits in international studies, particularly when initiated earlier in pregnancy [113,117]. Digital health is a promising but underdeveloped area. International evidence suggests that web-based platforms and telemedicine can improve fasting and postprandial glucose levels [118,119].

However, the Vietnamese intervention evidence remains methodologically limited. Most studies used pre–post designs without control groups, were conducted in major hospitals, and focused on short-term glycaemic outcomes. As a result, the evidence is insufficient to determine effectiveness, sustainability, scalability, or equity of these interventions. Future studies should use controlled designs, include longer follow-up, and evaluate outcomes beyond glycaemic stabilisation, including dietary adherence, maternal weight gain, medication use, birth outcomes, breastfeeding, postpartum glucose testing, and patient-reported outcomes.

### Policy and implementation implications

This review highlights several priorities for policy and practice. First, Vietnam’s national GDM guidelines require clearer pathways linking screening, diagnosis, counselling, treatment, and postpartum follow-up. Standardised screening, diagnostic criteria, and reporting are also needed to improve comparability, multicentre collaboration, and continuity of care.

Second, risk-stratified antenatal care could identify women needing earlier or more intensive support, particularly those with advanced maternal age, elevated BMI, family history of diabetes, adverse obstetric history, unhealthy dietary patterns, or physical inactivity.

Third, implementation should account for uneven service capacity across Vietnam’s public–private system. Standardised screening and basic counselling could be delivered at primary and commune levels, with referral to district or tertiary services for specialist care or pharmacotherapy.

Fourth, nutrition counselling should be culturally appropriate and food-based, moving beyond generic advice on sugar or starch restriction. For Vietnamese diaspora populations, care should also be linguistically accessible and adapted to Vietnamese dietary practices, with interpreter support where needed.

Fifth, family-inclusive education should be considered, as household beliefs and food practices influence women’s self-management.

Sixth, postpartum follow-up should be treated as a core component of GDM care because of the long-term risk of type 2 diabetes.

Finally, implementation research is needed to test scalable models such as group education, community-based counselling, digital health, and integrated postpartum prevention pathways, with relevance beyond Vietnam to other low- and middle-income and migrant health settings.

These priorities are relevant not only to Vietnam, but also to other low- and middle-income settings and migrant health systems seeking to respond to the growing burden of GDM.

### Research gaps

Despite increasing research on GDM in Vietnam, several important gaps remain.

First, epidemiological and surveillance: most studies were hospital-based and using heterogeneous diagnostic criteria, limiting comparability and national prevalence estimation.

Second, risk factor and mechanisms: evidence was largely descriptive, with limited multivariate analyses and little investigation of gene–environment, metabolomics or microbiome-related mechanisms.

Third, behavioural and psychosocial determinants: cultural beliefs, family support, dietary practices, and barriers to self-management remained underexplored.

Fourth, maternal and neonatal outcomes: most studies focused on short-term outcomes, with few longitudinal studies examining long-term maternal progression to type 2 diabetes or intergenerational effects in offspring.

Fifth, intervention evidence was limited to short, uncontrolled pre–post studies, with little research on postpartum diabetes prevention using either non-pharmacological or pharmacological approaches.

Finally, evidence among Vietnamese diaspora populations was sparse, and none evaluating interventions or culturally adapted care.

These gaps highlight the need for multicentre, longitudinal, and interdisciplinary research to inform evidence-based GDM prevention and management strategies.

### Limitations

This scoping review has several limitations. First, substantial heterogeneity in study design, diagnostic criteria, and sample characteristics limited comparability across studies and regions. Second, most studies were hospital-based and cross-sectional, potentially overrepresenting high-risk populations and limiting causal inference and temporal analysis. Third, publication and language bias cannot be excluded, as some grey literature and Vietnamese-language studies may not have been indexed in major databases. Fourth, incomplete methodological reporting in some studies, including sampling strategies and confounder adjustment, limited critical appraisal. Finally, prevalence estimates should be interpreted as descriptive rather than population-representative and reported risk factors should be considered candidate risk factors reflecting associations rather than confirmed causal relationships. Despite these limitations, this review provides a comprehensive synthesis of GDM research in Vietnam and highlights priorities for future multicentre and longitudinal studies.

## Conclusion

This scoping review mapped 76 studies on GDM among Vietnamese-origin women in Vietnam and diaspora settings. The available evidence indicated a substantial reported burden of GDM and identified recurring demographic, clinical, dietary, and lifestyle associations. Studies in diaspora populations generally reported a higher GDM prevalence among Vietnamese-origin women than among host-country populations; however, this evidence was limited and focused mainly on prevalence and selected obstetric outcomes.

Overall, the evidence base was fragmented, predominantly hospital-based, and methodologically heterogeneous. Key gaps remained in community-based surveillance, standardised diagnostic reporting, longitudinal maternal and offspring outcomes, psychosocial determinants, controlled interventions, postpartum prevention, and culturally responsive research involving Vietnamese diaspora populations. Future evidence generation should prioritise multicentre and community-based studies, longitudinal follow-up, robust intervention designs, and emerging areas such as sleep, digital health, metabolomics, genetics, and the gut microbiome. This evidence map provides a foundation for defining research priorities in Vietnam and Vietnamese migrant health settings.

## Supplementary Material

Supporting document PRISMA Checklist.docx

## Data Availability

No new data were generated. All data were obtained from cited published and grey literature.
